# ﻿Morphological and phylogenetic analyses reveal two new species of *Niesslia* (Niessliaceae, Hypocreales) from China

**DOI:** 10.3897/mycokeys.127.175675

**Published:** 2026-01-09

**Authors:** Xin Tian, Ming-Yi Zhang, Jian-Gan Hou, Yun-Jie Wu, Zhi-Yuan Zhang

**Affiliations:** 1 Guiyang Institute of Humanities and Technology, Guiyang 550025, China Guiyang Institute of Humanities and Technology Guiyang China; 2 College of Eco-Environmental Engineering, Guizhou Minzu University, Guiyang 550025, China Guizhou Minzu University Guiyang China; 3 School of Foreign Languages, Taiyuan University of Science and Technology, Taiyuan 030024, China Taiyuan University of Science and Technology Taiyuan China

**Keywords:** Asexual morph, fungal biodiversity, two new species, urban soils

## Abstract

The genus *Niesslia* is common on decaying plant and fungal substrates, as well as in soil. During a survey of culturable mycobiota in urban soils, six *Niesslia* isolates were obtained in Shanxi Province, China. Based on combined multigene phylogenetic analyses (ITS, LSU, *TEF1*, *TUB* and *ACT*) and morphological characteristics, two novel species, *Niesslia curvispora* and *N. shanxiensis*, are proposed. Detailed morphological descriptions, illustrations and phylogenetic analyses of the new taxa are presented.

## ﻿Introduction

*Niesslia* was introduced for a single species, *N. chaetomium* (Auerswald 1869). This genus is characterised by tiny, shiny, mostly dark brown and superficial perithecial ascomata, typically adorned with spines. A key feature is the presence of monocillium-like phialides, which are usually partly or entirely thick-walled under both *in situ* and culture conditions. Phialides sometimes extend from a widened, thick-walled base to a tapering conidiiferous neck, while others terminate in non-sporulating vesicles (Gams et al. 2019). Currently, there are 128 records of *Niesslia* in Index Fungorum (https://www.indexfungorum.org, accessed October 2025). According to Gams et al. (2019), *Niesslia* comprises 50 cultured species and approximately 40 species known only from herbarium specimens. However, some of the latter may need to be excluded from the genus (Gams et al. 2019). Recently, Hou et al. (2023) transferred *N. minutispora* out of *Niesslia*, designating it as the type species for the newly-established genus *Pseudoniesslia* and the new family Pseudoniessliaceae. Additionally, they revealed that *Niesslia* is polyphyletic (Hou et al. 2023).

Members of the genus *Niesslia* typically inhabit decaying plant and fungal substrates, including conifer twigs and needles, wood, grass leaves, lichens, polypores and rust fungi (Tanney et al. 2023). They can be saprotrophic, hypersaprotrophic or parasitic (Tsuneda and Hiratsuka 1980; Etayo et al. 2013; Ashrafi et al. 2017; Gams et al. 2019). This suggests that species may possess the ability to decompose xylan and cellulose or serve as potential biocontrol agents against plant pathogens, such as *N. mucida* and *N. nordinii* (Domsch and Gams 1969; Tsuneda and Hiratsuka 1980). Tanney et al. (2023) isolated a new species, *N. endophytica*, from black spruce (*Picea mariana*) needles. This fungus produced natural products with significant ecological functions and biological activities, particularly resorcylic acid lactones. Additionally, Ashrafi et al. (2017) observed that *N. gamsii* and *N. bulbillosa* parasitised the eggs of the cereal cyst nematode (*Heterodera filipjevi*), demonstrating potential for nematode biocontrol.

During our investigation of culturable mycobiota in urban soils in Shanxi Province, China, six isolates belonging to *Niesslia* were obtained. This study aimed to describe two new species, *N. curvispora* and *N. shanxiensis* and to provide detailed information for these species on substrate, molecular data availability, morphological characteristics and country of origin.

## ﻿Materials and methods

### ﻿Fungal isolation and morphological characterisation

In May 2025, the soil samples were collected from the green belts of Yingze Park and Taiyuan Zoo in Taiyuan City, Shanxi Province. Fungi were isolated and purified using the dilution method described by Pan et al. (2025). Pure colonies obtained on potato dextrose agar (PDA; Coolaber, China) were transferred on to fresh PDA, oatmeal agar (OA) and synthetic nutrient-poor agar (SNA) media (all from Coolaber, China). The cultures were subsequently incubated at 25 °C in darkness for 14 days to observe the macroscopic and morphological characteristics of the colonies. Morphological features were observed and recorded using a Zeiss Axio Imager A2 microscope and a Zeiss AxioCam MRc colour digital camera (Carl Zeiss Ltd., Munchen, Germany).

Dried culture (at 50 °C) specimens were deposited at the Fungarium (HMAS), Institute of Microbiology, Chinese Academy of Sciences (CAS). The ex-type strains for the novel species were deposited at the China General Microbiological Culture Collection Center (CGMCC), China. All living cultures were stored in a metabolically inactive state (i.e. kept in sterile 30% glycerol in a –80 °C freezer) and were deposited in the College of Eco-Environmental Engineering, Guizhou Minzu University. MycoBank numbers were registered for new names.

### ﻿DNA extraction, PCR amplification and sequencing

Total genomic DNA was extracted from living cultures using the BioTeke Fungal Genomic DNA Extraction Kit (DP2032, BioTeke, Beijing, China), adhering to the manufacturer’s protocol. Polymerase chain reaction (PCR) was performed to amplify the internal transcribed spacer region (ITS), large nuclear ribosomal subunit rDNA (LSU) region, translation elongation factor 1-α (*TEF1*), partial β-tubulin 2 (*TUB*) and partial γ-actin (*ACT*) genes. The amplification was carried out using the following primer pairs, respectively: ITS1/ITS4 (White et al. 1990), LR0R/LR5 (Vilgalys and Hester 1990; Rehner and Samuels 1994), EF1-983F/EF1-2218R (Rehner and Buckley 2005), Btub2Fd/Btub4Rd (Woudenberg et al. 2009) and ACT-512F/ACT-783R (Carbone and Kohn 1999). PCR was performed in a 25 µl reaction volume containing 2.0 μl genomic DNA extract, 1.0 μl each of forward and reverse primers, 8.5 μl double distilled water and 12.5 μl 2× MasterMix (Sangon Biotech, China). The PCR products were purified and sequenced by the same company (Sangon Biotech, Shanghai, China). All newly-generated sequences have been deposited in the GenBank database (Table 1).

**Table 1.** Strains used in this study, with information on the GenBank accessions of the sequences.

Species	Strains	GenBank accession numbers					References
		ITS	LSU	*TEF1*	*TUB*	*ACT*	
*Niesslia aemula*	CBS 556.75	MG827004	MG826805	MG896351	MG896302	MG896533	Gams et al. (2019)
*Niesslia aeruginosa*	CBS 264.89 T	MG826958	MG826762	MG896385	MG896258	MG896498	Gams et al. (2019)
Niesslia aff. barbula	CBS 692.94	MG827025	MG826830	MG896446	MG896325	MG896554	Gams et al. (2019)
*Niesslia albosubiculosa*	CBS 100348 T	MG826913	MG826718	MG896454	MG896220	MG896467	Gams et al. (2019)
*Niesslia allantoidea*	CBS 914.96 T	MG827040	MG826848	MG896453	MG896343	MG896567	Gams et al. (2019)
*Niesslia arctiicola*	CBS 476.80	MG826999	MG826802	MG896357	MG896297	MG896528	Gams et al. (2019)
*Niesslia arctiicola*	CBS 994.69 T	MG827044	MG826852	MG896384	MG896346	NA	Gams et al. (2019)
*Niesslia artocarpi*	CBS 582.73 T	MG827008	MG826809	MG896461	MG896306	MG896537	Gams et al. (2019)
*Niesslia aterrima*	CBS 388.85 T	MG826978	MG826781	MG896366	MG896277	MG896513	Gams et al. (2019)
*Niesslia aurantiaca*	CBS 158.72	MG826934	MG826739	MG896407	MG896237	MG896479	Gams et al. (2019)
*Niesslia barbula*	CBS 560.74	MG827005	MG826806	MG896434	MG896303	MG896534	Gams et al. (2019)
*Niesslia brevis*	CBS 125922 T	MG826929	MG826734	MG896387	MG896234	MG896476	Gams et al. (2019)
*Niesslia bulbillosa*	CBS 344.70 T	MG826971	MG826774	MG896360	MG896270	MG896507	Gams et al. (2019)
*Niesslia catenata*	CBS 694.70 T	MG827026	MG826831	MG896445	MG896326	MG896555	Gams et al. (2019)
Niesslia cf. gamsii	CBS 389.85	MG826981	MG826784	MG896359	MG896280	NA	Gams et al. (2019)
Niesslia cf. ilicifolia	CBS 390.70	MG826982	MG826785	MG896391	MG896281	MG896515	Gams et al. (2019)
*Niesslia cladii*	CBS 652.79 T	MG827020	MG826821	MG896365	MG896316	MG896548	Gams et al. (2019)
*Niesslia cladoniicola*	CBS 960.73 T	MG827042	MG826850	MG896370	MG896345	MG896569	Gams et al. (2019)
*Niesslia clarkii*	CBS 170.74 T	MG826936	MG826741	MG896378	MG896238	MG896481	Gams et al. (2019)
*Niesslia constricta*	CBS 760.69 T	MG827031	MG826839	MG896401	MG896334	MG896559	Gams et al. (2019)
*Niesslia cryptomeriae*	UESTCC 23.0245 T	OR887403	OR887113	PP076828	PP150073	PP150069	Tian et al. (2024)
*Niesslia curvisetosa*	CBS 660.94 T	NA	MG826823	MG896352	MG896318	MG896549	Gams et al. (2019)
***Niesslia curvispora***	**CGMCC 3.29401 T**	**PX413397**	**PX413403**	**PX422497**	**PX422503**	**PX422509**	**This study**
***Niesslia curvispora***	**ZY 25.002**	**PX413398**	**PX413404**	**PX422498**	**PX422504**	**PX422510**	**This study**
***Niesslia curvispora***	**ZY 25.003**	**PX413399**	**PX413405**	**PX422499**	**PX422505**	**PX422511**	**This study**
*Niesslia dimorphospora*	CBS 361.76	MG826975	MG826778	MG896348	MG896274	MG896511	Gams et al. (2019)
*Niesslia dimorphospora*	CBS 785.69 T	MG827035	MG826843	NA	MG896338	MG896563	Gams et al. (2019)
*Niesslia elymi*	CBS 607.75 T	MG827016	MG826817	MG896394	MG896312	MG896545	Gams et al. (2019)
*Niesslia endophytica*	DAOM 251709 T	OQ275026	NA	OQ295990	NA	NA	Tanney et al. (2023)
*Niesslia exigua*	CBS 152.68	MG826933	MG826738	NA	MG896236	MG896478	Gams et al. (2019)
*Niesslia exilis*	CBS 357.70	MG826972	MG826775	MG896368	MG896271	MG896508	Gams et al. (2019)
*Niesslia fuegiana*	CBS 368.77	MG826977	MG826780	NA	MG896276	MG896512	Gams et al. (2019)
*Niesslia fusiformis*	CBS 325.77 T	MG826969	MG826773	NA	MG896268	MG896505	Gams et al. (2019)
*Niesslia gamsii*	DSM 105458 T	MF681485	MF681496	MF681506	NA	NA	Ashrafi et al. (2017)
*Niesslia grisescens*	CBS 599.88 T	MG827013	MG826814	MG896396	MG896311	MG896542	Gams et al. (2019)
*Niesslia guizhouensis*	CGMCC 3.20780 T	OL897021	OL897063	ON568918	NA	ON568873	Zhang et al. (2023)
*Niesslia guizhouensis*	GZUIFR 21.913	OL897022	OL897064	ON568919	NA	ON568874	Zhang et al. (2023)
*Niesslia hennebertii*	CBS 389.70B T	MG826980	MG826783	MG896389	MG896279	NA	Gams et al. (2019)
*Niesslia hepworthiae*	CBS 153518 T	PV664940	PV664966	PV664036	PV664052	PV664004	Crous (2025)
*Niesslia heterophora*	CBS 150.70	MG826932	MG826737	NA	NA	NA	Gams et al. (2019)
*Niesslia ilicifolia*	CBS 459.74	MG826995	MG826798	MG896392	MG896293	MG896524	Gams et al. (2019)
*Niesslia indica*	CBS 313.74	MG826967	MG826771	MG896354	MG896266	MG896503	Gams et al. (2019)
*Niesslia leucoula*	CBS 101685	MG826916	MG826721	MG896362	MG896223	NA	Gams et al. (2019)
*Niesslia leucoula*	CBS 101741 T	MG826917	MG826722	MG896363	MG896224	NA	Gams et al. (2019)
*Niesslia libertiae*	CBS 153519 T	PV664941	PV664967	PV664037	PV664053	PV664005	Crous (2025)
*Niesslia ligustica*	CBS 697.86	MG827028	MG826833	MG896375	MG896328	MG896556	Gams et al. (2019)
*Niesslia ligustica*	CBS 684.95 T	MG826827	MF681502	MG896376	MG896322	MG896552	Gams et al. (2019)
*Niesslia loricata*	CBS 778.69 T	MG827034	MG826842	MG896388	MG896337	MG896562	Gams et al. (2019)
*Niesslia luzulae*	CBS 700.79	MG827029	MG826834	MG896350	MG896329	MG896557	Gams et al. (2019)
*Niesslia marinisedimenta*	SFC20240607-M014 T	PQ355567	PQ355586	PQ355490	PQ355470	NA	Lee et al. (2025)
*Niesslia mucida*	CBS 404.66 T	MG826989	MG826792	MG896420	MG896288	MG896519	Gams et al. (2019)
*Niesslia neoexosporioides*	CBS 146810 T	MW883432	MW883824	NA	MW890137	MW890027	Crous et al. (2021)
*Niesslia nieuwwulvenica*	CBS 148943 T	ON603771	ON603791	ON605629	ON605637	ON605620	Crous et al. (2022a)
*Niesslia nordinii*	CBS 101.63 T	MG826914	MG826720	MG896398	MG896222	MG896468	Gams et al. (2019)
*Niesslia pandani*	CBS 583.73 T	MG827009	MG826810	MG896358	MG896307	MG896538	Gams et al. (2019
*Niesslia parviseta*	KRAML-73346 T	OQ600193	OQ600191	OQ606818	NA	NA	Crous et al. (2023
*Niesslia phragmiticola*	CBS 149682 T	OQ628487	OQ629069	OQ627954	OQ627966	OQ627934	Crous et al. (2023)
*Niesslia physacantha*	CBS 474.74 T	MG826998	MG826801	MG896433	MG896296	MG896527	Gams et al. (2019)
*Niesslia podocarpi*	HKAS 131283 T	OR887404	OR887114	PP076829	PP150074	PP150070	Tian et al. (2024)
*Niesslia pseudoexilis*	CBS 148333 T	ON811511	ON811569	NA	ON803596	ON803514	Crous et al. (2022b)
*Niesslia rhizomorpharum*	CBS 642.85 T	MG827019	MG826820	MG896428	MG896315	MG896547	Gams et al. (2019)
*Niesslia rollhansenii*	CBS 686.74 T	MG827024	MG826828	MG896404	MG896323	NA	Gams et al. (2019)
***Niesslia shanxiensis***	**CGMCC 3.29402 T**	**PX413394**	**PX413400**	**PX422500**	**PX422506**	**PX422512**	**This study**
***Niesslia shanxiensis***	**ZY 25.005**	**PX413395**	**PX413401**	**PX422501**	**PX422507**	**PX422513**	**This study**
***Niesslia shanxiensis***	**ZY 25.006**	**PX413396**	**PX413402**	**PX422502**	**PX422508**	**PX422514**	**This study**
*Niesslia* sp.	CBS 493.73	MG827002	MG826804	MG896397	MG896300	MG896531	Gams et al. (2019)
*Niesslia* sp.	CBS 827.73	MG827037	MG826845	MG896364	MG896340	MG896565	Gams et al. (2019)
*Niesslia* sp.	CBS 885.73	MG827039	MG826847	MG896361	MG896342	NA	Gams et al. (2019)
*Niesslia sphaeropedunculata*	CBS 123802 T	MG826928	MG826733	MG896464	MG896233	NA	Gams et al. (2019)
*Niesslia stellenboschiana*	CBS 145531 T	MK876400	MK876441	NA	NA	NA	Crous (2019)
*Niesslia subiculosella*	CBS 326.77 T	MG826970	NA	MG896371	MG896269	MG896506	Gams et al. (2019)
*Niesslia tenuis*	CBS 432.66 T	MG826994	MG826797	MG896448	MG896292	MG896523	Gams et al. (2019)
*Niesslia tenuissima*	CBS 586.73A T	MG827010	MG826811	MG896427	MG896308	MG896539	Gams et al. (2019)
*Niesslia trachycarpi*	GZCC 21-0196 T	PP578084	PP621072	PP761000	PP816203	NA	Zhang et al. (2024)
*Niesslia waitemataensis*	CBS 324.77	MG826968	MG826772	MG896455	MG896267	MG896504	Gams et al. (2019)
*Niesslia xanthorrhoeae*	CBS 287.93 T	MG826961	MG826765	MG896383	MG896260	NA	Gams et al. (2019)
*Niesslia yunnanensis*	ZHKUCC 24-1061 T	PQ208502	PQ208504	NA	NA	NA	Dong et al. (2025)
*Niesslia yunnanensis*	ZHKUCC 24-1062	PQ208503	PQ208505	NA	NA	NA	Dong et al. (2025)
*Microascus trigonosporus*	CBS 218.31	MH855195	MH866643	HG380359	LM652655	NA	Vu et al. (2019)

Notes: T: Ex-type. NA, not available. DNA sequences for the new isolates were in bold.

### ﻿Phylogenetic analyses

Raw forward and reverse sequences were assembled using Lasergene software (version 6.0, DNASTAR). BLASTn search results showed that these sequences belonged to the genus *Niesslia*. Therefore, sequences were obtained from recently published data (Table 1; Gams et al. (2019); Crous et al. (2022a, b); Crous et al. (2023); Tanney et al. (2023); Zhang et al. (2023); Zhang et al. (2024)). Sequences alignments were generated using MAFFT v.7.037 (Katoh and Standley 2013) and trimmed using MEGA v.6.06 (Tamura et al. 2013). Concatenated sequences of ITS, LSU, *TEF1*, *TUB* and *ACT* were obtained using PhyloSuite v.1.2.3 (Xiang et al. 2023).

Phylogenetic analyses were inferred using both Bayesian and Maximum-Likelihood algorithms. The best evolutionary models for phylogenetic analyses were selected independently for each locus using ModelFinder (Kalyaanamoorthy et al. 2017) under the corrected Akaike Information Criterion (AICc). Maximum Likelihood (ML) analysis was conducted using the IQ-TREE v.1.6.11 (Nguyen et al. 2015) with 10,000 ultrafast bootstrap algorithm tests (Minh et al. 2013). Bayesian analyses were conducted with MrBayes v. 3.2 (Ronquist et al. 2012). The Markov Chain Monte Carlo (MCMC) method was used to perform 10^8^ simulations with a sampling frequency of 10^3^ generations and a 25% burn-in. Convergence and appropriate burn-in were assessed using Tracer v.1.5 (Drummond and Rambaut 2007).

## ﻿Results

### ﻿Phylogenetic analyses

A phylogenetic analysis of the genus *Niesslia* was conducted using a combined dataset of ITS, LSU, *TEF1*, *TUB* and *ACT* sequences. *Microascus trigonosporus* (CBS 218.31) was used as the outgroup, according to Tanney et al. (2023). The final alignment included 79 taxa representing 67 species and consisted of 2,558 nucleotide positions (ITS, 570 bp; LSU, 843 bp; *TEF1*, 483 bp; *TUB*, 386 bp; and *ACT*, 276 bp), including gaps. The best-fit model for the ML analysis was TIM2+F+I+I+R4 for ITS, TIM+F+R3 for LSU, GTR+F+I+I+R3 for *TEF1*, GTR+F+I+G4 for *TUB* and SYM+I+I+R4 for *ACT*. The best-fit model for the BI analysis was GTR+F+I+G4 for ITS, LSU and *TUB*, GTR+F+G4 for *TEF1* and SYM+I+G4 for *ACT*.

**Figure 1.**
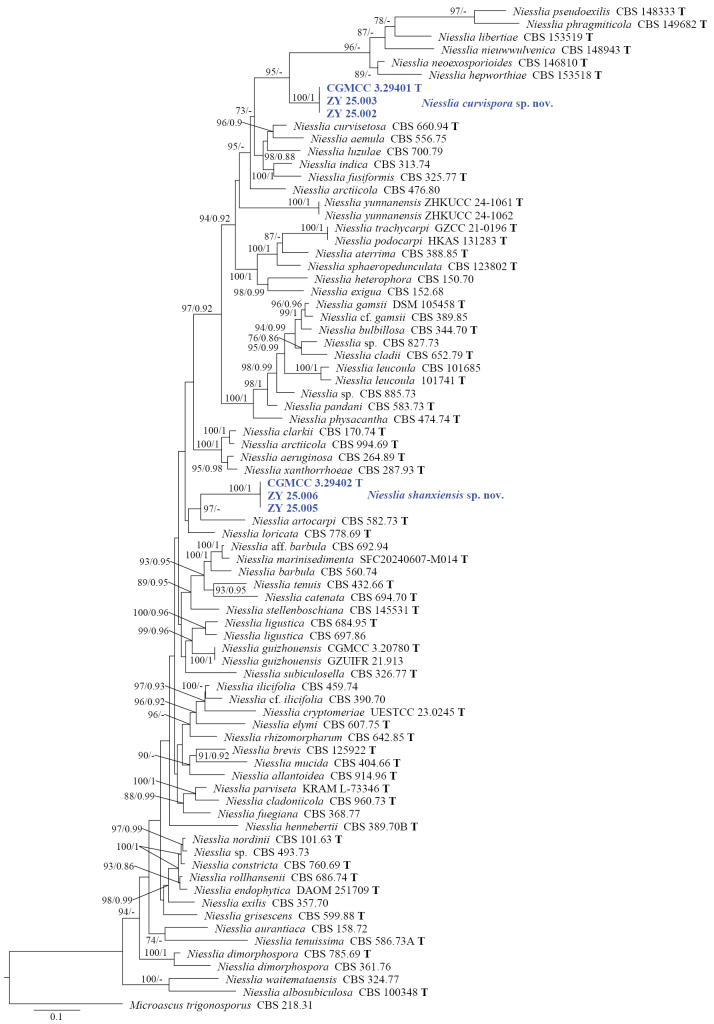
Phylogenetic tree inferred from a Maximum Likelihood analysis, based on a concatenated alignment of ITS, LSU, *TEF1*, *TUB* and *ACT* sequences from 79 isolates representing *Niesslia* and outgroup taxa. Numbers at branches indicate support values (IQ-TREE-BS/BI-PP) above 70%/0.90. The new species are printed in blue bold. Strains with a type status are indicated with “T”. The tree is rooted to *Microascus trigonosporus* (CBS 218.31). The scale bar represents the expected number of changes per site.

In the ML tree, the three isolates of *Niesslia curvispora* formed a highly supported (100%) subclade, which in turn clustered with high support (95%), together with a clade containing *N. hepworthiae*, *N. libertiae*, *N. neoexosporioides*, *N. nieuwwulvenica*, *N. phragmiticola* and *N. pseudoexilis* (Fig. 1). Similarly, the three isolates of *N. shanxiensis* formed a highly supported subclade (100%), which in turn clustered with strong support (97%), together with *N. artocarpi* (CBS 582.73) (Fig. 1). In the BI tree, the two new species proposed in this study each clustered as independent subclades with high support (100%), but their support for clustering with adjacent clades was low (Suppl. material 1). Although the support values derived from ML and BI analyses showed some variation, the phylogenetic tree topologies constructed by both methods were consistent (Fig. 1, Suppl. material 1). This result may be attributed to the following reasons: our analysis encompassed a larger number of species within the genus *Niesslia*, which is polyphyletic and exhibits abundant molecular variation amongst species.

### ﻿Taxonomy

#### ﻿ Niesslia curvispora

Taxon classificationFungiHypocrealesNiessliaceae

Ming.Y. Zhang & Zhi.Y. Zhang sp. nov.

D65EA4C0-52F0-532D-A2C9-AB9DFB5901E1

MB860790

Fig. 2

##### Type.

China • Shanxi, Taiyuan, Yingze Park 37.85°N, 112.56°E, soil, 17 May 2025, Zhi-Yuan Zhang (holotype HMAS 354201, dried culture; culture ex-type CGMCC 3.29401, *ibid*., ZY 25.001).

**Figure 2.**
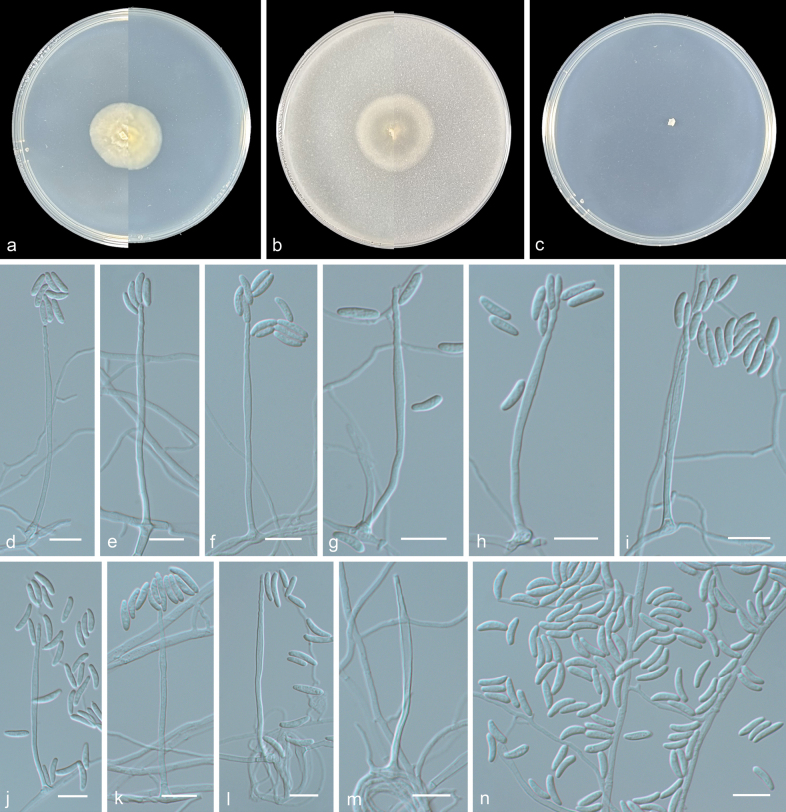
*Niesslia curvispora* (from holotype HMAS 354201). **a, b.** Upper and reverse views of cultures on PDA and OA after 14 days at 25 °C; **c.** Upper view of culture on SNA after 14 days at 25 °C; **d–m.**Monocillium-like conidiophores; **a, e, g, h.** Phialides proliferate percurrently with distinctly twisted or spiralled apex; **n.** Conidia. Scale bars: 10 μm (**d–m**).

##### Etymology.

The epithet “*curvispora*” refers to its curved conidia.

##### Description.

***Culture characteristics*** (14 days at 25 °C): Colony on PDA reaching 24–27 mm diam. thick, white, margin entire, reverse similarly coloured. Colony on OA reaching 27–29 mm diam. flat, grey, margin entire, reverse similarly coloured. No growth of observed on SNA.

***Mycelium*** consisting of hyaline, smooth-walled, branched, septate, 1–3 μm diam. hyphae. Sporulation abundant and phalacrogenous. ***Phialides*** usually monocillium-like with moderately thick-walled 1–2.5 μm wide basal portion, sometimes percurrently proliferating with a twisted or spiral apex and sometimes ending with a sterile vesicle, (37–)45–64 μm. ***Conidia*** adhering in slimy heads, aseptate, hyaline, smooth- and thin-walled, elongated-cylindrical, gradually tapering towards ends, hardly truncate at base, curved, 6.5–11.5 × 1.5–2.5 μm (av. 9.1 × 2.6 μm, n = 30). ***Chlamydospores*** and ***sexual morph*** not observed.

##### Geographical distribution.

Shanxi Province, China.

##### Additional material examined.

China • Shanxi, Taiyuan, Yingze Park 37.85°N, 112.56°E, soil, 17 May 2025, Zhi-Yuan Zhang, ZY 25.002. • Shanxi, Taiyuan, Taiyuan Zoo 37.91°N, 112.59°E, soil, 17 May 2025, Zhi-Yuan Zhang, ZY 25.003.

##### Notes.

Phylogenetic analysis showed that three new isolates (ZY 25.001–25.003) clustered in a single subclade with high support (100/1, Fig. 1). Morphologically, *Niesslia curvispora* shares curved conidia with *N. dimorphospora* and *N. rhizomorpharum* (Gams 1971; Gams et al. 2019). However, *N. curvispora* differs from *N. dimorphospora* by its shorter phialides (45–64 μm vs. 55–85 μm) and the shape of conidia (elongated-cylindrical vs. ellipsoidal) (Gams 1971; Gams et al. 2019). Additionally, *N. curvispora* differs from *N. rhizomorpharum* by the shape and size of its conidia (elongated-cylindrical, 6.5–11.5 × 1.5–2.5 μm in *N. curvispora* vs. elongate ellipsoidal, 4–7 × 1.5–2.0 μm in *N. rhizomorpharum*) (Gams et al. 2019). Phylogenetically, *N. curvispora* is related to a clade having *N. hepworthiae*, *N. libertiae*, *N. neoexosporioides*, *N. nieuwwulvenica*, *N. phragmiticola* and *N. pseudoexilis* (Fig. 1). However, *N. curvispora* differs from these species by its shorter phialides and curved conidia (Crous et al. 2021; Crous et al. 2022a, b, 2023; Crous 2025). In addition, based on a pairwise comparison of ITS, LSU, *TEF1*, *TUB* and *ACT*, *N. curvispora* (ex-type ZY 25.001) differs from *N. hepworthiae* (ex-type CBS 153518) by 7.9% (47/588, 16 gaps) in the ITS, 2.2% (19/846, two gaps) in the LSU, 68.8% (633/920, 443 gaps) in the *TEF1*, 27.8% (119/428, 44 gaps) in the *TUB* and 33.9% (106/312, 56 gaps) in the *ACT*; from *N. libertiae* (ex-type CBS 153519) by 9.9% (60/601, 24 gaps) in the ITS, 2% (17/843, one gap) in the LSU, 71.1% (622/874, 485 gaps) in the *TEF1*, 24.8% (90/362, 34 gaps) in the *TUB* and 31.7% (89/280, 24 gaps) in the *ACT*; from *N. neoexosporioides* (ex-type CBS 146810) by 6.3% (40/627, 17 gaps) in the ITS, 1.8% (16/846, two gaps) in the LSU, 25.4% (107/421, 37 gaps) in the *TUB* and 29.1% (88/302, 28 gaps) in the *ACT*; from *N. nieuwwulvenica* (ex-type CBS 148943) by 4.4% (25/561, seven gaps) in the ITS, 1.3% (11/805, one gap) in the LSU, 66.1% (578/874, 376 gaps) in the *TEF1*, 30.9% (119/385, 44 gaps) in the *TUB* and 24.7% (63/255, 20 gaps) in the *ACT*; from *N. phragmiticola* (ex-type CBS 149682) by 22.4% (125/556, 66 gaps) in the ITS, 5% (42/838, two gaps) in the LSU, 65.5% (584/891, 415 gaps) in the *TEF1*, 27.5% (112/407, 42 gaps) in the *TUB* and 35.1% (101/287, 45 gaps) in the *ACT*; from *N. pseudoexilis* (ex-type CBS 148333) by 24.6% (151/613, 93 gaps) in the ITS, 6.5% (52/800, one gap) in the LSU, 24.7% (99/400, 42 gaps) in the *TUB* and 70.3% (457/650, 390 gaps) in the *ACT*.

#### ﻿ Niesslia shanxiensis

Taxon classificationFungiHypocrealesNiessliaceae

Ming.Y. Zhang & Zhi.Y. Zhang sp. nov.

DF669341-9595-5D47-86E2-66981F18AED2

MB860791

Fig. 3.

##### Type.

China • Shanxi, Taiyuan, Taiyuan Zoo 37.91°N, 112.58°E, soil, 17 May 2025, Zhi-Yuan Zhang (holotype HMAS 354202, dried culture; culture ex-type CGMCC 3.29402, *ibid*., ZY 25.004).

**Figure 3.**
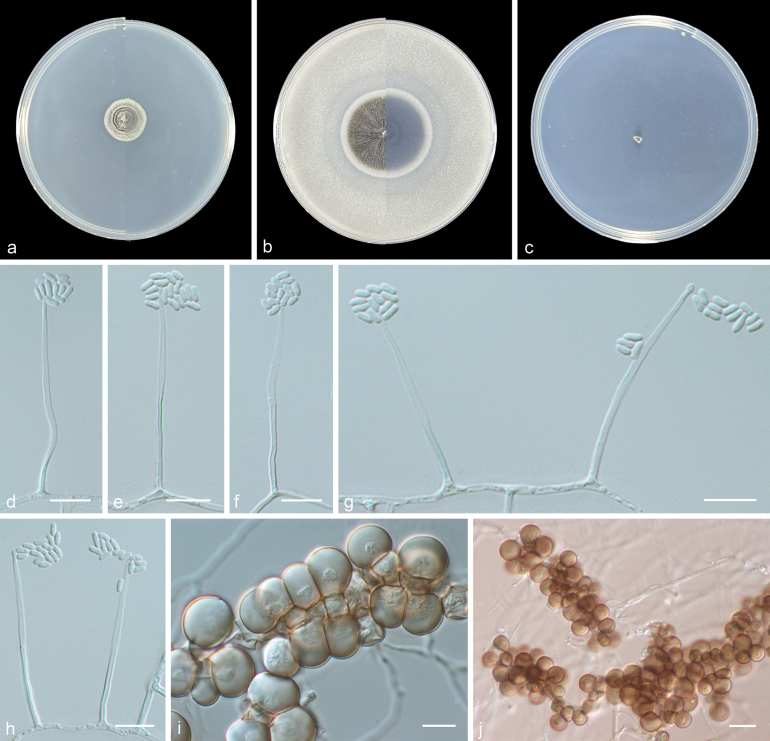
*Niesslia shanxiensis* (from holotype HMAS 354202). **a, b.** Upper and reverse views of cultures on PDA and OA after 14 days at 25 °C; **c.** Upper view of culture on SNA after 14 days at 25 °C; **d–h.** Conidiophores and conidia; **i, j.** Chlamydospores. Scale bars: 10 μm (**d–i**); 20 μm (**j**).

##### Etymology.

The epithet refers to the type location.

##### Description.

***Culture characteristics*** (14 days at 25 °C): Colony on PDA reaching 15–17 mm diam. powdery, have a circle of black wheel, grey to white from centre to margin, margin entire, reverse similarly coloured. Colony on OA reaching 34–35 mm diam. felted, drab to white from centre to margin, margin entire, reverse similarly coloured. No growth of observed on SNA.

***Mycelium*** consisting of hyaline, smooth-walled, branched, septate, 1–3 μm diam. hyphae. Sporulation abundant, phalacrogenous, with conidia adhering in slimy droplets, often coalescing. ***Phialides*** consisting of a thick-walled, 0.5–1.5 μm wide basal portion, extending into an elongate, slightly widened part up to 1.5–2 μm diam., total length 36–49 μm. ***Conidia*** adhering in slimy heads, aseptate, hyaline, smooth- and thin-walled, cylindrical with rounded ends, 2.5–5 × 1–2 μm (av. 3.6 × 1.4 μm, n = 30). ***Chlamydospores*** abundant, brown, smooth- and thin-walled, botryoidal, subglobose to globose, 11–16 μm. ***Sexual morph*** not observed.

##### Geographical distribution.

Shanxi Province, China.

##### Additional material examined.

China • Shanxi, Taiyuan, Yingze Park 37.85°N, 112.56°E, soil, 17 May 2025, Zhi-Yuan Zhang, ZY 25.005. • Shanxi, Taiyuan, Taiyuan Zoo 37.91°N, 112.59°E, soil, 17 May 2025, Zhi-Yuan Zhang, ZY 25.006.

##### Notes.

Phylogenetic trees showed that three new isolates (ZY 25.004–25.006) clustered in a single subclade with high support (100/1, Fig. 1). Morphologically, *Niesslia shanxiensis* share the presence of chlamydospores with *N. aeruginosa* and *N. loricata*. However, *N. shanxiensis* differs from *N. aeruginosa* in producing shorter phialides (36–49 μm vs. 40–65 μm) and larger chlamydospores (11–16 μm vs. 4.5–6 μm) (Gams et al. 2019). Additionally, *N. shanxiensis* differs from *N. loricata* by its cylindrical conidia and larger chlamydospores (11–16 μm in *N. shanxiensis* vs. 4.5–5.5 μm in *N. loricata*) (Gams 1971; Gams et al. 2019). Phylogenetically, *N. shanxiensis* is related to *N. artocarpi* (Fig. 1). However, *N. shanxiensis* can be distinguished from *N. artocarpi* by the shape and size of its conidia (cylindrical, 2.5–5 × 1–2 μm in *N. shanxiensis* vs. short-ellipsoidal, 2.2–3 × 1.3–1.5 μm in *N. artocarpi*) and by the presence of chlamydospores (Gams et al. 2019). In addition, based on a pairwise comparison of ITS, LSU, *TEF1*, *TUB* and *ACT*, *N. shanxiensis* (ex-type ZY 25.003) differs from *N. artocarpi* (ex-type CBS 582.73) by 11.5% (69/609, 29 gaps) in the ITS, 2.5% (22/847, three gaps) in the LSU, 4.5% (19/416, no gap) in the *TEF1*, 20.2% (83/410, 27 gaps) in the *TUB* and 27.5% (79/287, 23 gaps) in the *ACT*.

## ﻿Discussion

To date, the complete life cycle of many *Niesslia* species remains unclear, with only either the anamorph or teleomorph known for numerous taxa (Gams et al. 2019). Furthermore, some species rarely form ascomata under laboratory culture conditions, which severely constrains taxonomic studies and contributes to uncertainty in estimating species diversity within the genus. Consequently, members of *Niesslia* exhibit subtle morphological variations, making species delimitation virtually impossible without DNA barcoding and phylogenetic analysis (Gams et al. 2019). In the present study, we adhere to the taxonomic framework established by Gams et al. (2019), who revised the generic concept of *Niesslia* and provided a key to the accepted species. Two novel species, isolated from urban soils in Shanxi Province, China, *N. curvispora* and *N. shanxiensis*, were described, based on molecular phylogeny and morphological data. This study enhances our understanding of species diversity and geographical distribution within *Niesslia*.

It is perplexing that, although Gams et al. (2019) sequenced the ITS, LSU, *TEF1*, *TUB* and *ACT* from 126 strains representing over 40 species of *Niesslia*, they neither conducted phylogenetic analyses nor compared nucleotide sequence divergences. In accordance with the species delimitation criteria established by Jeewon and Hyde (2016), phylogenetic analyses should integrate the ITS region with at least one protein-coding gene and maintain a minimum nucleotide divergence of > 1.5% in the ITS regions between closely-related species. The two novel species described in this study, *N. curvispora* and *N. shanxiensis*, both demonstrate substantial sequence differentiation from their closest relatives (see notes for each species). In accordance with the work of Gams et al. (2019), we sequenced the ITS, LSU, *TEF1*, *TUB* and *ACT* sequences of the isolated strains and conducted phylogenetic analyses. The results indicate that these molecular markers can effectively distinguish species within the genus *Niesslia*.

Recently, Hou et al. (2023) confirmed the polyphyletic of the genus *Niesslia*, based on multi-locus (LSU, ITS and *rpb2*) phylogenetic analyses. However, their phylogenetic reconstruction suffered from insufficient sampling of *Niesslia*, encompassing only a small number of species within the genus. This study, together with several recent studies (Tanney et al. 2023; Zhang et al. 2023), reveals the diversity and species richness of the genus. Therefore, future research should expand the sampling scope to include data from as many *Niesslia* species as possible, thereby further clarifying the appropriate taxonomic status of species within this genus.

## Supplementary Material

XML Treatment for Niesslia curvispora

XML Treatment for Niesslia shanxiensis

## ﻿Additional information

### Use of AI

No use of AI was reported.

### Author ORCIDs

Xin Tian https://orcid.org/0009-0008-4880-9454

Ming-Yi Zhang https://orcid.org/0009-0000-0691-3941

Jian-Gan Hou https://orcid.org/0000-0002-2502-8742

Yun-Jie Wu https://orcid.org/0009-0005-1316-5546

Zhi-Yuan Zhang https://orcid.org/0000-0003-2031-7518

### Data availability

Sequence data are available in GenBank (see Table 1).

## Supplementary materials

Supplementary material 1 Phylogenetic tree Data type pdf https://binary.pensoft.net/file/1508796This dataset is made available under the Open Database License (http://opendatacommons.org/licenses/odbl/1.0/). The Open Database License (ODbL) is a license agreement intended to allow users to freely share, modify, and use this Dataset while maintaining this same freedom for others, provided that the original source and author(s) are credited.Xin Tian, Ming-Yi Zhang, Jian-Gan Hou, Yun-Jie Wu, Zhi-Yuan Zhang
