# Supplementary figures and images for: ﻿Morphological and phylogenetic analyses reveal two new species of Niesslia (Niessliaceae, Hypocreales) from China
Source: MycoKeys. 2026 Jan 9;127:73–87. doi: 10.3897/mycokeys.127.175675 (PMC12811755; doi:10.3897/mycokeys.127.175675)

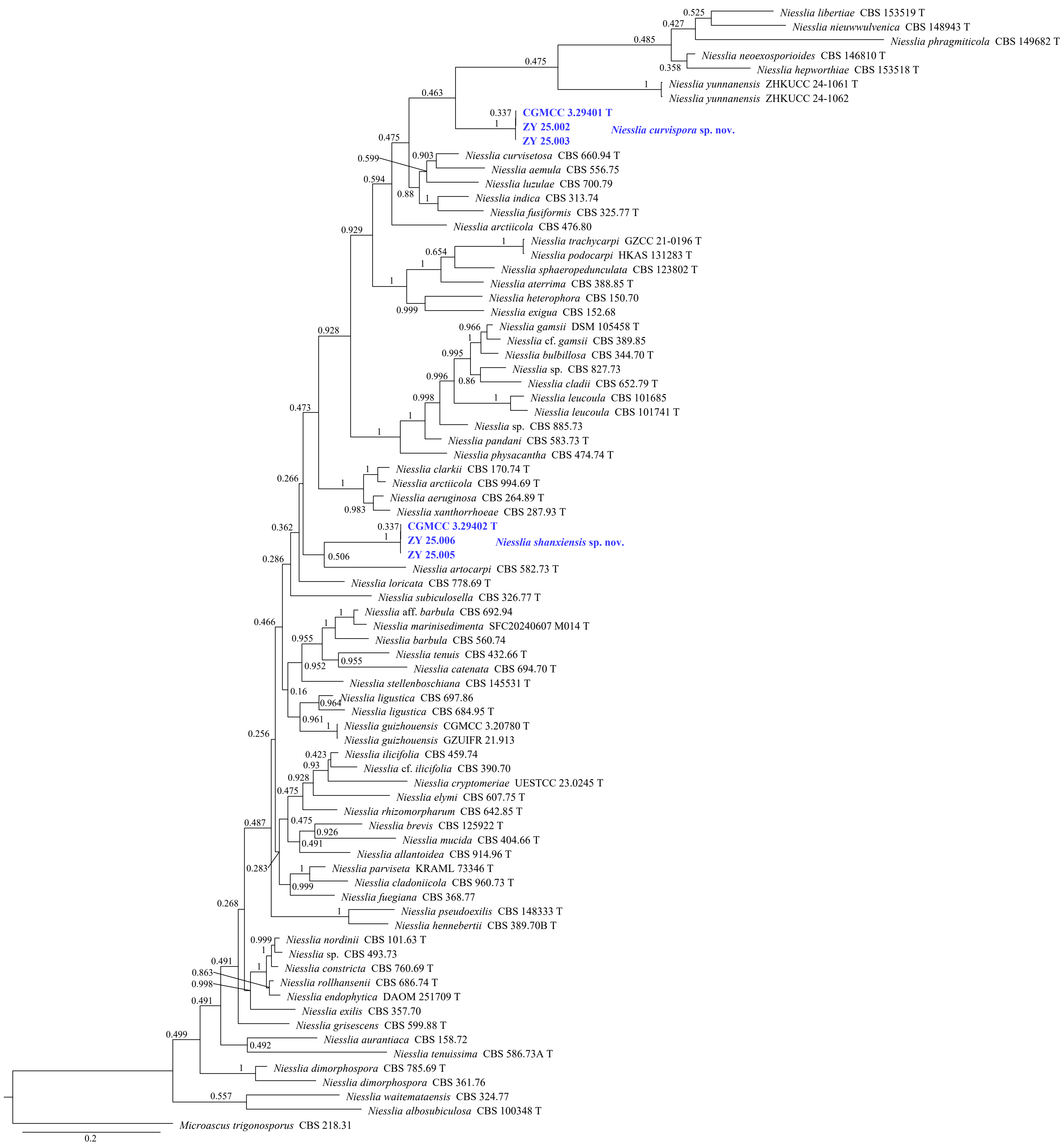

Supplement: Supplementary material 1 — Phylogenetic tree [file mycokeys-127-073-s001.pdf]
